# Soluble membrane receptors, interleukin 6, procalcitonin and C reactive protein as prognostic markers in patients with severe sepsis and septic shock

**DOI:** 10.1371/journal.pone.0175254

**Published:** 2017-04-05

**Authors:** Juan-Jesús Ríos-Toro, Mercedes Márquez-Coello, José-María García-Álvarez, Andrés Martín-Aspas, Ricardo Rivera-Fernández, Ana Sáez de Benito, José-Antonio Girón-González

**Affiliations:** 1 Intensive Care Unit, Hospital Serranía de Ronda, Málaga, Spain; 2 Infectious Unit, Hospital Universitario Puerta del Mar, Facultad de Medicina, Universidad de Cádiz, Instituto de Investigación e Innovación en Ciencias Biomédicas de Cádiz (INiBICA), Cádiz, Spain; 3 Biochemistry, Hospital Universitario Puerta del Mar, Facultad de Medicina, Universidad de Cádiz, Instituto de Investigación e Innovación en Ciencias Biomédicas de Cádiz (INiBICA), Cádiz, Spain; Azienda Ospedaliero Universitaria Careggi, ITALY

## Abstract

**Background:**

The objective of this study was to explore the diagnostic and prognostic value of soluble triggering receptor expressed on myeloid cell 1 (sTREM-1), soluble cluster of differentiation 14 (sCD14), soluble cluster of differentiation 163 (sCD163), interleukin-6 (IL-6), procalcitonin (PCT), and C-reactive protein (CRP) serum levels for patients with severe sepsis and septic shock in an intensive care unit (ICU).

**Methods:**

Fifty patients admitted at the ICU with the diagnosis of severe sepsis or septic shock were studied. SOFA and APACHE II scores as well as serum biomarkers were measured at days 0, 2 and 5. The influence of these variables on 28-day mortality was analyzed. Twenty healthy individuals served as controls.

**Results:**

Baseline serum concentrations of sTREM-1, sCD163, IL-6 and PCT correlated with SOFA score. Only sTREM-1 levels correlated with APACHE II score. The 28-day mortality rate for all patients was 42%. The absence of risk factors for infection, presence of septic shock, baseline values of sCD14 and decrease of PCT and IL-6 from baseline to day 5 were variables associated to mortality in the univariate analysis. The unique independent factor associated to mortality in the multivariate analysis was a decrease of PCT higher than 50% from days 0 to 5.

**Conclusions:**

Serum levels of sTREM-1 are correlated with the severity of sepsis. A 50% decrease of PCT was the unique variable associated with survival in the multivariate analysis.

## Introduction

Sepsis is an important medical problem with significant morbidity and mortality [1]. From a clinical perspective, sepsis is defined and diagnosed based on the observation of physiopathological changes in response to an infection, i.e. the interaction between microbes and the human immune system (both innate and acquired), the synthesis of mediators, and the response of the organism [1].

During the last decade, much effort has been directed toward the identification of biomarkers that are useful in the differential diagnosis of sepsis and in the prediction of prognosis [2–14]. A biomarker is an analyte that can be used to assess a normal or pathological process, or the response to a therapeutic intervention. In general, sepsis biomarkers can be classified based on their role as mediators of systemic inflammation: 1) Molecules expressed on the membrane of phagocytes [Triggering receptors expressed on myeloid cells-1 (TREM-1), CD14 receptor, CD163 receptor]. 2) Biomarkers can also be proinflammatory cytokines, such as interleukin (IL)-6. 3) Acute phase proteins, such as procalcitonin (PCT) or C reactive protein (CRP).

Triggering receptors expressed on myeloid cells-1 (TREM-1) are antigens of the immunoglobulin superfamily detected on the cell membrane of neutrophils and monocytes [2]. Expression is activated as a response to bacteria and fungi [3]. The CD14 receptor, located on the cell membrane of polymorphonuclear cells, monocytes and macrophages, among others, recognizes the bacterial lipopolysaccharide of Gram negative bacteria, as well as other bacterial products (peptidoglicans, spirochetal lipoprotein) [4,5]. The monocyte receptor CD163, the only type of hemoglobin scavenger receptor specifically expressed on the macrophage membrane, participates in monocyte-endothelium interactions [6] and in the inhibition of the T lymphocyte response [7]. It has been demonstrated to play a role in inflammation [8]. After interacting with their ligands, these molecules promote the expression of proinflammatory cytokines, such as IL-6, and contribute to the systemic inflammatory response [5]. Each of these molecules can be detected as plasma soluble receptors (sTREM-1, sCD14 or presepsin, sCD163) [9–11]. An increased shedding of these receptors is due to infectious/inflammatory stimuli, and they may be valuable diagnostic parameters for monitoring macrophage activation in inflammatory conditions. Although their physiologic function continues to be discussed, an antiinflammatory role has been proposed [9–11].

IL-6 is synthesized mainly by monocytes and macrophages. It stimulates adrenocorticotropic hormone (ACTH) synthesis, B cell differentiation, and cytotoxic T cell activation and is one of the main factors responsible for the liver synthesis of acute-phase proteins [12]. IL-6 stimulates the synthesis and secretion of several proteins or decrease their catabolism. Among these, procalcitonin (PCT) levels increase in the presence of proinflammatory cytokines, such as IL-1, IL-6, or tumor necrosis factor α (TNF-α), probably due to the inhibition of PCT proteolysis [13]. PCT modulates calcium metabolism, regulates cytokine and nitric oxide synthesis, and probably modulates the response to pain [13]. C reactive protein (CRP) is a liver-derived plasma protein; its concentration significantly increases in inflammatory processes in response to IL-6 [14]. CRP aggregates with damaged cells, apoptosis fragments, and bacterial, fungal, or parasitic components [14]. After aggregation, CRP is recognized by the C1q complement fraction and activates the classic complement pathway [14].

The usefulness of these biomarkers in the differentiation of a non-infectious systemic inflammatory response and sepsis has been previously demonstrated [15–19]. The accuracy of several of these markers in the prognosis of sepsis has also been analyzed [14,20–28]. Whereas the results about the prognostic value of sTREM-1 are controversial [20–22], sCD14 has been demonstrated to be useful in the diagnosis and prognosis of severe sepsis and septic shock [23,24]. sCD163 has been studied in the diagnosis and prognosis of bacteremia; however, the specificity of this molecule is low [25,26]. Serum concentrations of IL-6 have been correlated with the mortality of septic patients [27]. With reference to PCT, it has been established that PCT-guided therapy may reduce antibiotic exposure without increasing the mortality rate in primary care, emergency department, and intensive care unit (ICU) settings [28]. Finally, CRP is a useful marker of evolution of inflammatory processes, although its specificity is limited [14].

The objective of the present work is to assess these biomarkers in the evaluation of the severity of severe sepsis and septic shock and to perform a comparison with well-established scores, such as APACHE II and SOFA. Also, the prognostic usefulness of baseline values and the timely modifications of serum concentrations of these molecules will be analyzed.

## Patients and methods

This single-center prospective observational study was performed from January 1, 2014, through January 31, 2015, in the 16-bed mixed intensive care unit (ICU) of the “Serranía de Ronda” Hospital, Malaga, Spain. The “Serranía de Ronda” Hospital is a 300-bed non-academic teaching hospital. All specialties except neurosurgery and cardiac surgery are available. The hospital has an ICU-based medical emergency team.

All consecutive patients admitted to the ICU, more than 18 years old, with a diagnosis of severe sepsis/septic shock according the definitions of the Sepsis Survival Campaign 2012 [29] were included. If a patient had more than one ICU episode in the study period, only the first episode was included in the study. Exclusion criteria were: 1) previous immunodeficiency, either congenital, human immunodeficiency virus infection or hematological malignancies; 2) blood transfusion in the previous three months, because this could modify serum levels of the studied molecules; 3) treatment with corticosteroids or immunomodulators in the previous six months. If a patient had neither systemic inflammatory response syndrome (SIRS) nor sepsis, the patient was excluded from the study.

Twenty healthy controls, in which the absence of evidence of diseases was evaluated by clinical history and laboratory studies, served as controls.

### Study schedule

Diagnostic criteria followed the definitions of the Survival Sepsis Campaign 2012 [29]. The diagnosis of sepsis was based on the SIRS criteria in the presence of a known infection, and the diagnosis of severe sepsis in cases of sepsis-induced tissue hypoperfusion or organ dysfunction. In the case of maintained arterial hypotension without a response to volume infusion, necessitating the administration of vasoactive drugs, a diagnosis of septic shock was made.

The following variables were collected upon admission: 1) age and sex; 2) risk factors for infection (chronic obstructive pulmonary disease, diabetes mellitus, antibiotic treatment in the previous three months, central intravascular catheter, bladder catheter, active neoplasia–hematologic malignancies were excluded-); if the patient has no one of these risks factors of infection, the denomination „absence of risk factors of infection”was used; 3) previous antibiotic therapy; 4) site of infection; 5) severity of infection. Sepsis-related Organ Failure Assessment (SOFA) [30] and Acute Physiology and Chronic Health Evaluation II (APACHE II) [31] scores were measured upon admission based on laboratory data and medical records.

Because it has been demonstrated that acute kidney insufficiency (AKI) is associated with increased concentrations of sTREM-1 [32], the development of AKI was considered among independent variables. AKI was defined according to the 2012 KDIGO criteria [33]: a 48-hour absolute increase in serum creatinine of at least 26.4 μmol/L, and an increase of at least 50% from baseline that is known or presumed to have occurred within the prior 7 days, and a decline in urine output to not more than 0.5 mL/kg per hour for at least 6 hours.

Patients were treated according the guidelines of the Survival Sepsis Campaign 2012 [29]. Antibiotic therapy was considered adequate if the isolated microbia were sensitive to the antibiotic used in the empirical treatment [34]. Patients were followed until hospital discharge or death.

Blood samples were collected upon admission and after two and five days of hospitalization by venipuncture. After centrifugation, the plasma was stored at -80°C until analysis. Plasma concentrations of sTREM-1, sCD14, sCD163 and IL-6 were measured with specific sandwich enzyme-linked immunosorbent assays (R&D systems, Minneapolis, USA). PCT was measured by a time resolved amplified cryptate emission assay (Kryptor PCT, Brahms Diagnostica, Berlin, Germany). CRP was measured by a inmunoturbidometry assay (CardioPhase hsCRP; Siemens Healthcare Diagnostics, Deerfield, IL, USA).

### Statistical analysis

Quantitative variables are given as median (25–75 interquartile interval; IQR). Qualitative variables are presented as absolute number and percentage. Because quantitative variables were not normally distributed, non parametric tests were used to compare them. Comparisons between quantitative variables were performed using the Mann Whitney U test for independent data and Wilcoxon’s test for paired data. Categorical variables were compared with the chi-square test with Yates’ correction, when indicated. Correlations among variables were assessed using Spearman’s test.

Biomarkers concentration changes, using serum levels at day 0 as the reference, were calculated according to the following formula:

100x(serumlevelofbiomarkeratday0–serumlevelofbiomarkeratday2or5)/serumlevelofbiomarkeratday0

Mortality at 28 days of hospital stay was the dependent variable. Characteristics of survivors vs. non-survivors were compared using univariate analysis. Receiver operating characteristics (ROC) curves were used to discriminate between survivors and non-survivors based on percentage of change in serum levels of biomarkers from day 0 to day 5 of ICU stay and the areas under the ROC curves (AUCs) were determined. The outcome variable was 28-day mortality.

A multivariate Cox regression analysis (Wald method) was performed to determine those factors independently associated with mortality. Variables with a p value less than 0.05 after the univariate analysis were included in the multivariate analysis. The start date for the study was the day of admission. The last date was the day of death.

For statistical analysis, we used SPSS 18.0 (SPSS Inc., Chicago, IL). All statistical tests were two-tailed. A p value < 0.05 was considered significant.

### Ethics

Approval for the study was granted by the Ethics and Medical Research Committee and the Malaga Provincial Committee. Patients or their representatives provided written informed consent to participate in this study.

## Results

### Patient characteristics upon admission

The general characteristics of the patients and healthy controls are shown in Table 1.

**Table 1:** General characteristics of the patients.

		Healthy controls (n = 20)	Severe sepsis/Septic shock patients (n = 50)
**Age (years)**		65 (54–70)	68 (53–75)
**Gender male (%)**		70	72
**Risk factors of infection (%)**			
	Absent	100	28
	Chronic obstructive pulmonary disease		18
	Diabetes mellitus		22
	Neoplasia		4
	Bladder catheter		36
	Central venous catheter		30
	More than 1 risk factor		42
**Site of infection (%)**			
	Respiratory		34
	Urinary		8
	Abdominal		48
	Central nervous system		2
	Cardiovascular		2
	Soft tissues and bone		2
	Unknown		4
**Bacteriemia (%)**			56
	Primary bacteriemia		2
	Secondary bacteremia		54
**Nosocomial origin (%)**			30
**Previous antibiotic therapy (%)**			34
**SOFA score**			7 (1–16)
**APACHE II score**			19 (6–43)
**Septic shock (%)**			66
**Serum lactate (mg/dl)**			19 (13–32)
**Urgent surgery (%)**			30
**Mechanical ventilation (%)**			52
**Cathecolamin therapy (%)**			72
**Acute renal insufficiency (%)**			42

Upon admission, the serum concentration of each biomarker was significantly higher than that detected in healthy controls (Table 2). Serum levels of sTREM-1, IL-6, PCT, and CRP were significantly higher in patients with septic shock than in those with severe sepsis (Table 2). Patients with bacteremia showed higher serum concentrations of CRP [242 (156–292) vs 152 (60–147) mg/l, p = 0,017) and PCT [6,0 (1,3–29,3) vs 1,0 (0,7–12,3) ng/ml, p = 0,013] than those without it. The rest of biomarkers did not shown statistical differences among patients with or without bacteremia (data not shown).

**Table 2:** Serum concentrations of biomarkers in patients with severe sepsis/septic shock and healthy controls.

	Healthy controls (n = 20)	Severe sepsis/Septic shock patients (n = 50)				
		Overall (n = 50)	P vs healthy controls	Severe sepsis (n = 17)	Septic shock (n = 33)	P severe sepsis vs septic shock
**sTREM-1 (pg/ml)**	135 (98–152)	1334 (784–2004)	<0,001	814 (378–1322)	1766 (1150–2468)	<0,001
**sCD14 (100 x ng/ml)**	28 (23–31)	51 (15–77)	<0,001	51 (22–66)	48 (15–90)	0,875
**sCD163 (ng/ml)**	368 (101–664)	1279 (805–1962)	<0,001	1266 (686–1922)	1363 (958–1981)	0.479
**Interleukin 6 (pg/ml)**	2 (0–3)	62 (11–139)	<0,001	24 (7–63)	81 (14–167)	0.010
**Procalcitonin (ng/ml)**	0,05 (0,02–0,09)	2,3 (0,5–20,8)	<0,001	0.7 (0.5–2.2)	13.2 (1.3–31.7)	0.001
**C reactive protein (mg/l)**	40 (12–55)	221 (110–281)	<0,001	133 (46–230)	247 (153–292)	0.007

Twenty-one patients developed AKI. Serum concentrations of sCD163 and sTREM-1 were significantly higher in patients who developed AKI than in the rest of individuals [sCD163, 1840 (1322–2103) vs 904 (663–1447) ng/ml, p < 0,001; sTREM-1, 1982 (1354–2760) vs 1050 (634–1424) pg/ml, p = 0,002]. The rest of biomarkers did not shown statistical differences among patients with or without AKI (data not shown).

The SOFA score was significantly correlated with serum concentrations of sTREM-1, sCD163, IL-6, and PCT in the overall group of patients. The APACHE II score was significantly correlated with serum levels of sTREM-1 (Table 3). Correlations of sTREM-1 and SOFA or APACHE II scores were detected both in patients who developed AKI (SOFA and sTREM-1, r = 0,618, p = 0,004; APACHE II and sTREM-1, r = 0,479, p = 0,010) and in those without AKI developpement (SOFA and sTREM-1, r = 0,471, p = 0,015; APACHE II and sTREM-1, r = 0,581, p = 0,002).

**Table 3:** Correlations among SOFA and APACHE II scores and serum concentrations of studied biomarkers in the overall group of patients.

	SOFA score		APACHE II score	
	r	p	r	p
**sTREM-1 (pg/ml)**	0,505	< 0,001	0,487	0,001
**sCD14 (100 x ng/ml)**	0,067	0,654	-0,078	0,501
**sCD163 (ng/ml)**	0,293	0,046	0,215	0,146
**Interleukin 6 (pg/ml)**	0,316	0,030	0,270	0,077
**Procalcitonin (ng/ml)**	0,478	0,001	0,280	0,056
**C reactive protein (mg/l)**	0,251	0,082	0,287	0,052

Note: Correlations among variables were assessed using Spearman’s test

Blood cultures enabled the isolation of bacteria or fungi in 28 patients (Table 4).

**Table 4:** Bacteria isolated in blood cultures of patients with severe sepsis/septic shock.

Bacteria		Severe sepsis/Septic shock patients (n = 50)
**Gram positive cocci (n,%)**		3 (6%)
	*Streptococcus pneumoniae*	1 (2)
	*Staphylococcus aureus*	1 (2)
	Staphylococcus coagulasa neg	1 (2)
**Gram negative bacilli (n, %)**		14 (28%)
	*Escherichia coli*	9 (18)
	*Enterobacter aerogenes*	2 (4)
	*Klebsiella pneumoniae*	1 (2)
	*Kluivera* spp	1 (2)
	*Proteus mirabilis*	1 (2)
**Anaerobes (n, %)**		1 (2)
	*Bacteroides fragilis*	1 (2)
**More than 1 bacteria (mixed infections) (n, %)**		8 (16)
	*Escherichia coli + Streptococcus* spp	1 (2)
	*Escherichia coli + Enterococcus faecium*	1 (2)
	*Escherichia coli + Bacteroides* spp *+ Enterococcus* spp	2 (4)
	*Escherichia coli + Bacteroides* spp *+ Streptococus* spp	1 (2)
	*Proteus penneri + Enterococcus faecium*	1 (2)
	*Alcaligenes* spp *+ Enterococcus* spp	1 (2)
	*Pseudomonas aeruginosa + Enterococcus faecium*	1 (2)
**Fungi (n, %)**		2 (4)
	*Candida albicans*	2 (4)
**Negative blood cultures (n,%)**		22 (44)

### Evolution of patients during hospitalization

Besides urgent surgery, mechanical ventilation, or renal replacement therapy, if indicated, all patients received antibiotic therapy. Taking into account the sensitivity of isolates, antibiotic therapy was considered adequate in 39 patients (78% of cases).

During their time in the ICU, 21 patients (42%) died. The median time of hospitalization was 9 (IQR, 3–28) days in those who died vs. 23 (IQR, 10–28) days in those who survived (p < 0.001).

**Fig 1:**
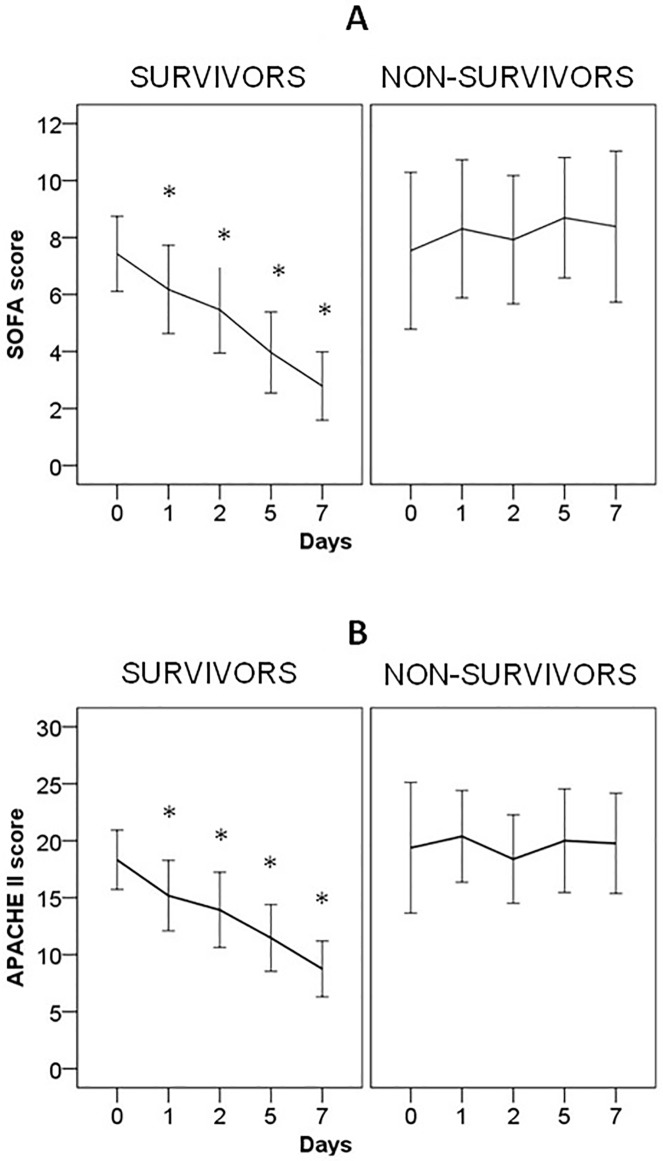
Changes of SOFA (A) and APACHE II (B) scores of patients with severe sepsis/septic shock during the first 7 days of ICU stay. Patients are classified in two groups: survivors and non-survivors. Data are shown as mean (95% confidence interval). Survivors shown significant decreases of SOFA and APACHE II scores from day 0 to the followed days (*, p < 0,001). In contrast, no significant difference of SOFA and APACHE II scores was detected in non-survivors patients in the days of ICU stay.

Changes in the SOFA and APACHE II scores in patients who survived vs. those who died during the ICU stay are shown in Fig 1.

**Fig 2:**
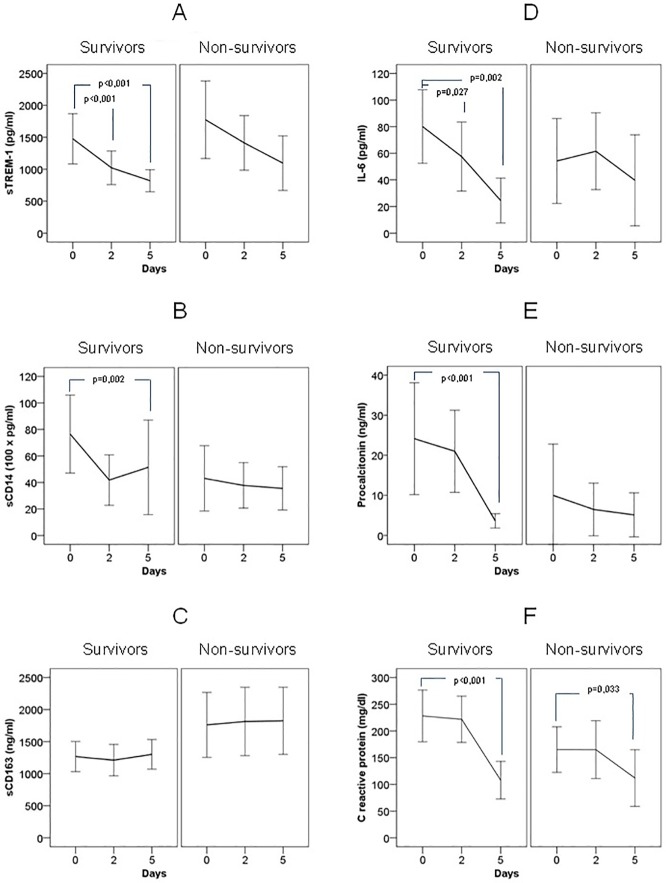
Changes of serum concentrations of sTREM-1 (A), sCD14 (B), sCD163 (C), IL-6 (D), procalcitonin (E) and C reactive protein (F) of patients with severe sepsis/septic shock during the first 5 days of ICU stay. Patients are classified in two groups: survivors and non-survivors. Data are shown as mean (95% confidence interval). Significant differences in sTREM-1, IL-6, procalcitonin and C reactive protein from day 0 to day 5 of ICU stay were detected in survivors.

A significant decrease in serum concentrations of sTREM-1, sCD14, and IL-6, but not of those of sCD163, PCT, and CRP, was detected in patients who survived from day 0 to day 2. At day 5, a significant decrease in the concentrations of sTREM-1, sCD14, IL-6, PCT, and CRP, but not of those of sCD163, with reference to serum levels of day 0, was detected in survivors. In contrast, there was no significant difference in the serum levels of these biomarkers taken at the different time points in patients who died (Fig 2). However, when the differences in the change of biomarker concentrations between survivors and non-survivors were compared, only the differences in IL-6 and PCT levels from day 0 to day 5 reached statistical significance (Table 5).

**Table 5:** Changes in serum levels of biomarkers during the first 5 days of intensive care unit stay in patients who survive and those who died with severe sepsis/septic shock.

Biomarker		Survivors	Non survivors	p
**sTREM-1 (pg/ml) (% change)**				
	Difference Day 0-Day 2	-28 (-47; -7)	-6 (-23; +3)	0,085
	Difference Day 0-Day 5	-38 (-60; +14)	-33 (-59; +11)	0,720
**sCD14 (100 x ng/ml) (% change)**				
	Difference Day 0-Day 2	-59 (-80; +5)	-10 (-92; +115)	0,584
	Difference Day 0-Day 5	-70 (-88; +30)	+17 (-60; +216)	0,087
**sCD163 (ng/ml) (% change)**				
	Difference Day 0-Day 2	-4 (-18; +10)	+2 (-3; +27)	0,148
	Difference Day 0-Day 5	-5 (-24; +35)	+2 (-14; +27)	0,795
**Interleukin 6 (pg/ml) (% change)**				
	Difference Day 0-Day 2	-28 (-73; +18)	-2 (-50; +92)	0,130
	Difference Day 0-Day 5	-86 (-98; -44)	+4 (-81; +116)	0,027
**Procalcitonin (ng/ml) (% change)**				
	Difference Day 0-Day 2	-27 (-43; +19)	+22 (-32; +90)	0,070
	Difference Day 0-Day 5	-78 (-92; +57)	-29 (-75; +68)	0,007
**C reactive protein (mg/dl) (% change)**				
	Difference Day 0-Day 2	-4 (-25; +18)	-5 (-21; +40)	0,878
	Difference Day 0-Day 5	-67 (-82; +7)	-19 (-77; +21)	0,457

Notes

1. Modifications of concentrations of biomarkers, taken as reference the serum levels at day 0, were obtained according the following formula: 100 x (serum level of biomarker at day 0 –serum level of biomarker at day 2 or 5) / serum level of biomarker at day 0

2. Changes at day 2 and 5 are expressed as percentages of baseline serum concentrations detected at day 0. Patients are divided in groups according to the survival outcome as survivors (n = 29) and non-survivors (n = 21)

**Fig 3:**
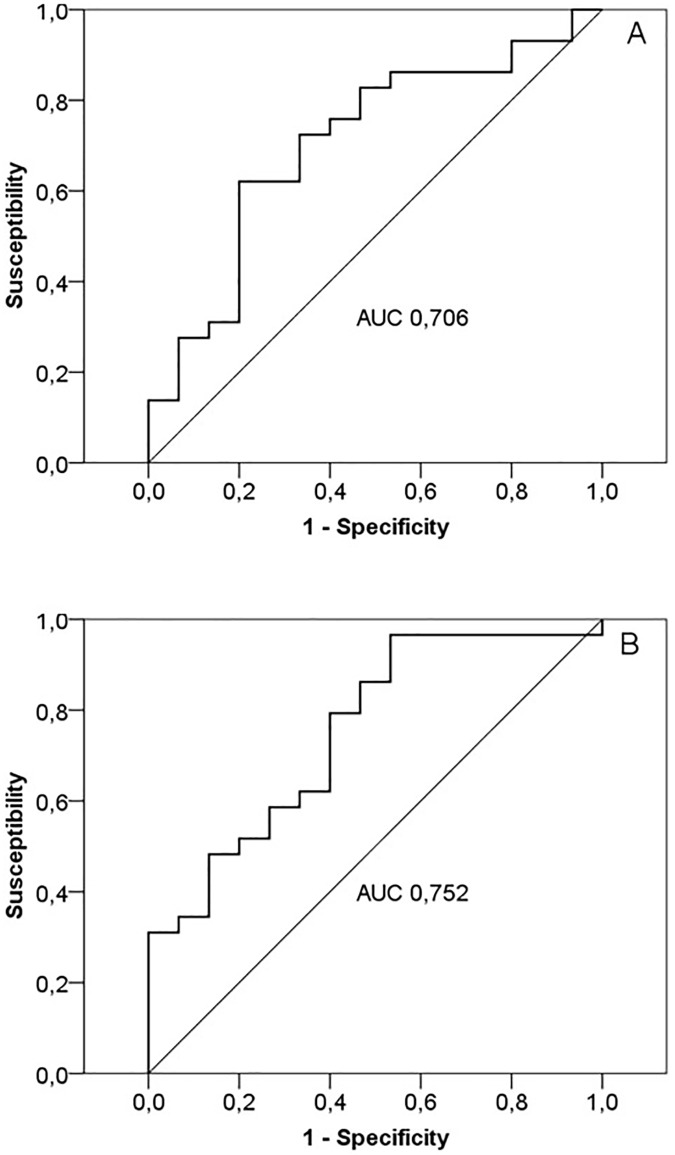
Receiver operating characteristics (ROC) curves used to discriminate survivors compared to non-survivors with severe sepsis/septic shock based on percentage of change in serum levels of IL-6 (A) and procalcitonin (B) from day 0 to day 5 of ICU stay.

The area under the ROC curves which relate survival with modifications in serum levels of IL-6 and PCT from day 0 to day 5 (Fig 3) was 0.706 and 0.752, respectively. The sensitivity and specificity of modifications in IL-6 or PTC in relation to survival as a function of different cutoff points are shown in Table 6. A cutoff point of 50%, i.e. a 50% decrease in the serum concentrations of PCT and IL-6 from day 0 to day 5, was selected. Differential characteristics among survivors and patients who died are shown in Table 7.

**Table 6:** Sensitivity and specificity of modifications in percentage of interleukin 6 or Procalcitonin in relation to survival in function of different cutoff points in patients with severe sepsis/septic shock.

Biomarker	% decrease of serum levels from day 0 to day 5	Sensitivity (%)	Specificity (%)	Positive predictive value for survival (%)	Negative predictive value for survival (%)
**Interleukin 6**	25%	83	57	73	71
	50%	76	71	79	68
	75%	62	76	78	59
	100%	21	95	86	47
**Procalcitonin**	25%	90	62	77	81
	50%	83	72	77	74
	75%	55	81	80	57
	100%	3	100	100	43

**Table 7:** Differential characteristics of patients who survive and those who died with severe sepsis/septic shock.

Variable		Survivors (n = 29)	Non survivors (n = 21)	p
**Age (years)**		66 (48–74)	71 (63–78)	0,079
**Gender male (%)**		76	67	0,534
**Absence of risk factor for infection (%)**		52	86	0,016
**Site of infection (%)**				0,297
	Respiratory	28	43	
	Abdominal	55	38	
	Urinary	10	5	
	Others	7	5	
	Unknown	0	10	
**Nosocomial infection (%)**		21	43	0,123
**Previous antibiotic therapy (%)**		33	35	1,000
**Bacteremia (%)**		62	48	0,391
**SOFA score**		7 (5–11)	7 (4–12)	0,414
**APACHE II score**		18 (14–23)	19 (15–26)	0,195
**Septic shock (%)**		65	67	0,017
**Acute kidney insufficiency (%)**		35	57	0,152
**Baseline serum lactate (mg/dl)**		17 (13–31)	19 (13–34)	0,853
**Baseline sTREM-1 (pg/ml)**		1226 (706–1914)	1562 (974–2250)	0,375
**Baseline sCD14 (100 x ng/ml)**		58 (22–105)	24 (11–55)	0,030
**Baseline sCD163 (ng/ml)**		1266 (720–1823)	1578 (981–2352)	0,143
**Baseline IL-6 (pg/ml)**		62 (12–156)	44 (10–92)	0,431
**Baseline procalcitonin (ng/ml)**		8,1 (0,7–27,1)	1,2 (0,4–9,2)	0,092
**Baseline C reactive protein (mg/dl)**		146 (137–286)	159 (86–240)	0,281
**Urgent surgery (%)**		35	24	0,537
**Mechanical ventilation (%)**		48	57	0,578
**Cathecolamin therapy (%)**		66	81	0,341
Adequate antibiotic therapy (%) *		89	70	0,315

* It was considered only in those cases of bacteremia demonstrated (survivors 18 cases; non survivors, 10 cases)

**Fig 4:**
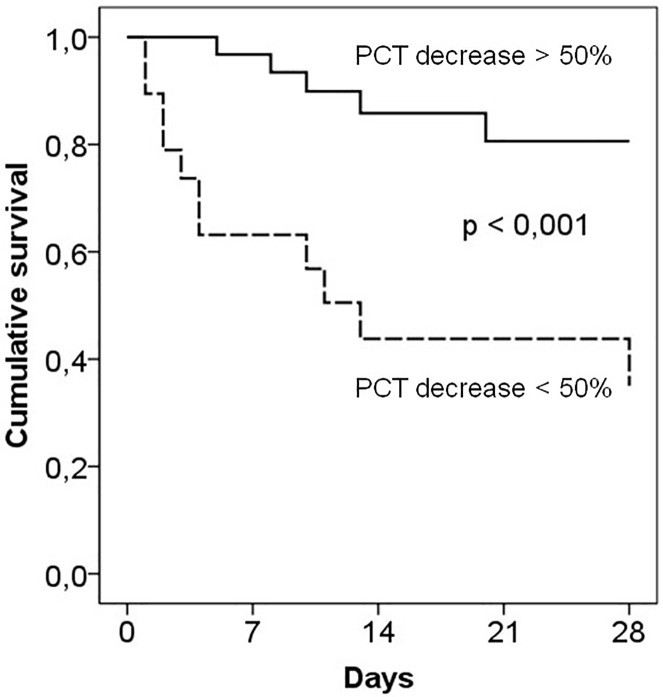
Kaplan Meier curves of survival of patients with severe sepsis/septic shock classified in function of a decrease of procalcitonin levels higher (continuous line) or lower (dashed line) than 50% from day 0 to 5 of ICU stay. A significant difference was detected among both groups of patients.

A multivariate analysis (Cox regression), including those variables with statistical significance in the previous bivariate analysis and differences in the serum concentrations of IL-6 and PCT between days 0 and 5 (a 50% decrease in concentration was selected in each case) was performed. Only a reduction > 50% of the baseline level in the PCT concentration from day 0 to day 5 was associated with survival [Exp (B) 4.47], confidence interval 95% 1.72–11.63, p = 0.002). Kaplan Meier survival curves as a function of the decrease in the PCT level from day 0 to 5 is shown in Fig 4; only 29% of patients with a decrease < 50% in PCT from day 0 to day 5 survived at day 28 compared to an 86% of those with a decrease > 50%.

## Discussion

This work has analyzed the accuracy of several molecules as biomarkers of severity and prognosis of patients with severe sepsis/septic shock. The biomarkers analyzed in this study are representative of different steps in the physiopathology of sepsis: recognition of the antigen by monocytes/macrophages (sTREM-1, sCD14), monocyte-endothelium interactions (sCD163), synthesis of proinflammatory cytokines (IL-6), and the response of target organs to these cytokines (PCT and CRP).

Using SOFA and APACHE II as accepted scores of the severity of sepsis [30,31], a significant correlation has been demonstrated between these scores and serum levels of some analyzed biomarkers, such as sTREM-1, IL-6, and PCT. Especially appealing was the assessment of sTREM-1, because it was the only molecule significantly correlated with both the SOFA and APACHE II scores. This is a previously demonstrated finding; effectively, serum sTREM-1 levels are significantly different in patients with sepsis compared to those with severe sepsis/septic shock [35–37] and these levels were correlated with the SOFA score [38]. Moreover, a correlation between sepsis severity and serum levels of IL-6 [39] and PCT [23,37,40–42] had been observed previously.

However, the severity assessment value of sCD14 (presepsin) and sCD163 was limited in our study. Other studies [39,43,44] have shown that presepsin levels are significantly associated with different ICU parameters reflecting the intensity and severity of severe sepsis/septic shock (APACHE II and SOFA score, days of intensive care treatment, days of mechanical ventilation, and days of catecholamine treatment), although other authors have also failed to demonstrate higher levels in patients with septic shock compared with those with sepsis [24]. Two possible explanations can be formulated. 1) Because sCD14 is cleaved from the monocyte/macrophage-specific CD14 receptor complex after binding with lipopolysaccharides (LPS) and LPS binding protein (LPB) during systemic infections [45], it is logical to consider that sCD14 levels will be dependent of the frequency of Enterobacteria isolation, so levels could be different as function of the responsible flora. However, Endo et al. observed that serum levels of sCD14 were similar in patients with bacteremia by Gram negative and Gram positive bacteria [46]. 2) The level of presepsin decreased rapidly after treatment [47]. In our series, a 34% of patients had received antibiotic therapy, which may have modified serum levels of sCD14.

In contrast with sCD14, previous studies have also failed to demonstrate an association between sCD163 and sepsis severity [36]. Both IL-6 and IL-10 stimulate, whereas LPS and interferon-γ decrease, the expression of CD163 molecules on the surface of macrophages [6, 48]. Also, hemolysis, a non-exceptional process in severe sepsis/septic shock, influences the expression of the CD163, the scavenger receptor of hemoglobin [49]. Therefore, the serum concentration of sCD163 is considered to be affected by the balance of different stimuli, resulting in higher or lower expression of the CD163 receptor; this aspect was not studied in this work.

It was also demonstrated that CRP failed to reflect sepsis severity, a previously published finding [50]. Because serum concentrations of CRP does not distinguish infectious from non-infectious inflammatory response [51,52], CRP could be considered just an inflammatory biomarker [14].

Mortality in our series was a 42%. The SOFA and APACHE II scores in the non-surviving group tended to increase over time, while they decreased gradually in the surviving group. Parameters assessed upon ICU admission that were significantly different between survivors and non-survivors in the univariate analysis were the presence of a risk factor for infection (probably because of a greater suspicion and early initiation of treatment against sepsis in these cases), septic shock, and a higher concentration of sCD14. There was no significant difference in age, the proportion of patients with bacteremia or adequate antibiotic therapy, or SOFA and APACHE II scores. Previous articles have assessed to these characteristics present at baseline which define to those patients with a poor prognosis. Thus, a higher APACHE II or SOFA score [53,54], the presence of bacteremia [55], or inadequate antibiotic therapy [56] have been implicated in the prognosis. Also, a higher concentration of biomarkers has been considered to have prognostic significance, as indicative of greater activation of the inflammatory and vasoactive systems [22,23,39]. However, implementing those measures indicated in the Surviving Sepsis Campaign [29], especially early surgical treatment if indicated, immediate antibiotic therapy, adequate and timely volume restitution, and initiating catecholamine treatment, the clinical course of patients could be better and, at least in part, independent of baseline characteristics [57,58]. In fact, the unique factor associated with survival was the significant decrease in the serum concentration of PCT. It has been previously established that the changes of PCT serum levels might guide antimicrobial therapy in patients with pneumonia [59–61] or in those admitted to the ICU with a bacterial infection [28,62].

Importantly, a prolonged time (5 days) was needed to obtain significant differences in the PCT concentration from baseline levels. Effectively, the serum concentration of PCT did not significantly change in the first 48 hours in our work, probably as a consequence of the dynamics of PCT synthesis and secretion: after a systemic bacterial infection, PCT levels increase in the first 2–3 hours and reach a peak at 12–48 hours [63].

Patients in which the PCT levels markedly decreased by >50% by day 5 survived better than those in which PCT concentrations dropped less (survival at day 28, 86% and 28%, respectively). Because the baseline levels of biomarkers did not discriminate among survivors and non-survivors, it is important to monitor changes in the concentration and not only the absolute values obtained at each time point. Moreover, taking into account the diverse variables that can influence mortality, the performance of a multivariate analysis is a key aspect to detect those parameters independently associated with death. These are two different aspects of this work when compared to other studies that have demonstrated the importance of PCT (or other biomarkers) as a prognostic variable [38–41,43]. Also, it is remarkable that PCT values represent the host response after a bacterial insult. Thus, progressive elevations in PCT levels indicate that the expression of PCT is continuously increasing and that more pro-inflammatory cytokines and mediators are being released in the body. In contrast, an adequate down-regulation of these systems after the initial reaction against the infection is a favorable prognostic marker. That is, it is not the microbe, or the initial innate immune response to these bacteria, but the host response to immune mediators, such as cytokines, that predicts outcomes.

### Limitations

This study was performed at a community hospital, including medical and surgical patients. Thus, the conclusions obtained can be applied to this type of patient and hospital. The isolation of multi-resistant microbes is infrequent in our hospital. Consequently, empiric antibiotic therapy to suspected microorganisms is usually adequate. Finally, the sample size was only 50 patients, but all patients completed the follow-up. This allowed us to complete the objectives of this work.

## Conclusions

Considering the SOFA and APACHE II scores as being indicative of the severity of sepsis, the correlation between baseline serum sTREM-1 levels and these scores make possible it to consider sTREM-1 as a biomarker of sepsis severity. During the evolution of sepsis, 42% of patients died. No baseline variables were associated with survival in the multivariate analysis. A 50% decrease in PCT from day 0 to day 5

## Abbreviations

APACHE II: acute physiology and chronic health evaluation
CD14: cluster of differentiation 14, presepsin
CD163: cluster of differentiation 163
I**CU**: intensive care unit
i.e.: in essence
IQR: interquartile interval.
IL-6: interleukin 6.
PCT: procalcitonin.
CRP: C reactive protein.
ROC: receiver operating characteristics.
SIRS: systemic inflammatory response syndrome.
SOFA: sepsis-related organ failure assessment.
TREM-1: triggering receptors expressed on myeloid cells-1.
TNF alpha: tumor necrosis factor alpha.
